# Supplementary material for: Measuring, visualizing, and diagnosing reference bias with biastools
Source: Genome Biol. 2024 Apr 19;25:101. doi: 10.1186/s13059-024-03240-8 (PMC11027314; doi:10.1186/s13059-024-03240-8)
Supplement: Supplementary file 1 — Additional file 1: Figure S1. Full normalized mapping balance to normalized assignment balance (NMB-NAB) plot. Figure S2. Normalized mapping balance to normalized assignment balance (NMB-NAB) plot stratified by allele length. Figure S3. Example of local decision by Bowtie 2 and BWA MEM. Figure S4. Example of local decision by default BWA MEM and BWA MEM with option -L 30. Figure S5. An example of the low coverage result of LevioSAM 2 and direct-to-GRC methods. Table S1. Number of balanced sites and different categories of biased sites on chromosome 16. [file 13059_2024_3240_MOESM1_ESM.docx]

**1^st^ round**

**Reviewer 1**

The authors have presented a comprehensive work on reference bias study, introducing a strongly formalized approach for its detection, evaluation, and visualization. Furthermore, the manuscript is well written and explains rigorously how biastools work. As authors also write, what reference bias means and how it happens has received little attention. I totally agree and this work is a great contribution, given that what was missing was precisely a formalization of the reference bias problem. Regarding this issue, usually, what is reported are just plots showing variant length versus allele frequency relative to read alignment bias. Only recently something different was published (https://doi.org/10.1093/bioinformatics/btac743), related to how to achieve unbiased graphical representations of pangenomes with the variation graph model.

The authors have confirmed that end-to-end alignments are better than local alignments in reducing reference bias and explained why. Going in the opposite way, can they suggest possible optimizations to apply to current aligners' implementations? Reference bias is an issue for the downstream analysis, but it is also expensive, as aligners try to align as much as possible until they eventually give up. Can totally novel alignment algorithms be conceived now that we formally know that end-to-end alignment is better than local alignment?

Going back to pangenomic graphs, I suppose the authors have created the graphs with VG CONSTRUCT, starting from the reference genome and VCF files. I can't find this information in the manuscript. It would be interesting to know how graphs made with other methods would perform when it comes to reference bias. I think about the two main ways to get base-level resolved pangenome graphs nowadays: Minigraph-Cactus (https://doi.org/10.1038/s41587-023-01793-w) and PGGB (https://doi.org/10.1101/2023.04.05.535718). It would also be nice to have an automated way to highlight the regions of the graphs that show more of the reference bias problem.

How biastools could help in the polishing process of aligning the raw sequences reads against the de novo assembly made with the same reads? Assembly curation might take advantage of the tools.

Figure 2: why have authors reported results only about chromosome 20? I agree that it is a comfortable chromosome for development and benchmarking, as it is small enough and an "easy" one, but I would like to see how reference bias affects complex genomic regions as well, like those on chromosome 16, which has one of the highest levels of segmentally duplicated sequence among the human autosomes (https://doi.org/10.1038/nature03187), and/or the short arms of the acrocentric chromosomes, that were resolved for the first time very recently (https://doi.org/10.1126/science.abj6987) and are highlighting interesting heterologous recombination in the human pangenome (https://doi.org/10.1038/s41586-023-05976-y).

Typo on Page 6, Line 52: outliers are colored light gray instead of colored light green as reported.

Can I see another Figure that is Figure 2, but stratifying by INDELs length? How does the plot change with INDELs longer than 10/50bps?

Figures 3 and 4: Why are insertions more affected by reference bias? I see this type of plot all the time, where the insertions have a little more variance with respect to the deletions. Is it because reads carrying insertions are more likely to align incorrectly? Why?

About the unphased GIAB HG002 VCF, can't authors try to phase those variants and repeat the analysis? There are lots of HiFi reads for HG002, for example, that can be used for the phasing. This would also allow authors to quantify the expected inaccuracy in using the unphased VCF.

In the scan mode evaluation, authors reported that "60% of SNV sites and 67% of gap sites called biased by the NMB-NAB analysis fell inside regions called biased by scan mode." I was hoping for a bit higher percentages. Have authors investigated the cases that do not fall inside bias regions to understand which characteristics those errors have (for example, sequence content, GC%, local sequence entropy, etc…)? Hopefully, authors should just check and incorporate just a few more properties to be able to catch more biased regions without foreknowledge of HET locations.

Biastools works on datasets from diploid individuals. What about the plans to extend it in order to work in a haploid setting? And polyploidy? Why did authors restrict to diploidy only?

The work shows biastools usage with short reads. Can I already apply biastools on long-read alignments in its current state?

Probably, it is out of the scope, but how much reference bias affects alignment coming from an all-vs-all comparison of reads/contigs?

Both reference numbers 13 and 29 have issues with the name "Jouni Sirén".

**Reviewer 2**

The authors present biastools, a tool for analyzing reference bias in genomic sequencing data. It addresses the known problem in genomic data analysis where reads from non-reference alleles might fail to align correctly to their point of origin. This software can be of interest to the bioinformatic community, allowing users and developers of bioinformatics software to tune and optimize alignment algorithms as well as downstream tools.

Major comments.
1. Providing performance metrics of the tool, such as CPU time and memory usage, would be useful for potential users.
2. The discussion mentions that "biastools's methods are applicable to long-read alignments as well." It would be nice to add the validation of this statement, especially considering that long reads are actively used for modern whole-genome assemblies. It is in particular challenging with short reads alone to assess highly repetitive regions such as centromeres in T2T-CHM13 (discussed in 2.4). Also worth noting, T2T-CHM13 assembly contains other long complex repeats besides centromeres that might be worth taking into account for the calculations for Table 4.
3. The proposed approach to the quality assessment is interesting, but its validation lacks a bit of clarity. For example, page 14: "scan mode called 1,676 biased regions on chromosome 20, covering 1.8% of its bases". In total, 447 SNVs and 115 gaps fell inside biased regions, but it is unclear how many of the 1,676 biased regions do not contain gaps or SNVs. Would be good to have information where these regions are located and why they were called "biased".
4. The study would benefit from a comparison with other assembly quality evaluation tools, or at least a clear explanation of how biastools differs in scopes and application from some other reference-based tools. While the direct assessment of "reference bias" is quite unique, there are many different tools for assembly quality assessment providing various informative metrics. Such explanation and comparison with known methods could help readers to better understand how they could use biastools in their practice.

Minor comments.
1. Authors could consider the possibility to extend the aligners' comparison with minimap2 as one of the most widely used aligners.
2. The paper contains minor typos and syntax errors. For instance, page 19, "the the individual". Page 16, "We collect all the bias regions improved by 25% in LevioSAM 2, can measure how many improved bases are from the regions near centromere, and how many are not." - this sentence seems to be a bit unclear.
2. It would be convenient to provide a small test dataset that can be used by new users to familiarize with the software.

**Authors’ response**

Reviewer #1: The authors have presented a comprehensive work on reference bias study, introducing a strongly formalized approach for its detection, evaluation, and visualization. Furthermore, the manuscript is well written and explains rigorously how biastools work. As authors also write, what reference bias means and how it happens has received little attention. I totally agree and this work is a great contribution, given that what was missing was precisely a formalization of the reference bias problem. Regarding this issue, usually, what is reported are just plots showing variant length versus allele frequency relative to read alignment bias. Only recently something different was published (https://doi.org/10.1093/bioinformatics/btac743), related to how to achieve unbiased graphical representations of pangenomes with the variation graph model.

We thank the reviewer for their careful reading of the paper and we added a citation in the Discussion section to the "Unbiased pangenome graphs" paper as an example of where the field is moving in the future.

The authors have confirmed that end-to-end alignments are better than local alignments in reducing reference bias and explained why. Going in the opposite way, can they suggest possible optimizations to apply to current aligners' implementations? Reference bias is an issue for the downstream analysis, but it is also expensive, as aligners try to align as much as possible until they eventually give up. Can totally novel alignment algorithms be conceived now that we formally know that end-to-end alignment is better than local alignment?

Our work does show that end-to-end alignment effectively reduces reference bias for a particular class of bias event: i.e. the "loss" bias event for gaps. However, we would not argue based on our results in this manuscript that end-to-end alignment is strictly preferred. For example, soft clipping is effective at ensuring that a greater proportion of reads align in cases where the reads have high error rates at one end.

To highlight this point and to clarify our view, we added new text to the Discussion, which can be found at the end of the second paragraph starting with the sentence "Findings obtained using Biastools will help in designing the next generation of reference representations and alignment algorithms…"

Going back to pangenomic graphs, I suppose the authors have created the graphs with VG CONSTRUCT, starting from the reference genome and VCF files. I can't find this information in the manuscript.

We agree that this needed clarification. We added a new Methods subsection titled "Construction of pangenome graphs" that provides these details, including our use of the vg autoindex" command to create the graphs.

It would be interesting to know how graphs made with other methods would perform when it comes to reference bias. I think about the two main ways to get base-level resolved pangenome graphs nowadays: Minigraph-Cactus (https://doi.org/10.1038/s41587-023-01793-w) and PGGB (https://doi.org/10.1101/2023.04.05.535718).

In principle, biastools can be used to compare any two methods, whether they differ in the alignment algorithm used, the reference representation used, or both.

However, we think it is outside the scope of this manuscript to perform comparisons where the only variable is the method used to construct the graph. While we certainly expect to find differences between the graph representations, the interpretation of those differences would require detailed discussion of how different graph representations work, which is outside our scope.

It would also be nice to have an automated way to highlight the regions of the graphs that show more of the reference bias problem.

Again, we think this is outside the scope of the current manuscript, which is not exclusively concerned with graph representations. Biastools is equally applicable to linear, graph pangenome, or non-graph pangenome alignment methods. We contend that new experiments focusing on how biastools results can be used to diagnose issues with pangeonme graph structures is best explored in future work focused more exclusively on graphs.

How biastools could help in the polishing process of aligning the raw sequences reads against the de novo assembly made with the same reads? Assembly curation might take advantage of the tools.

We would say that this is not a use case for biastools. Biastools is geared toward characterizing reference bias with particular attention to the way bias manifests when reads come from a donor who is unrelated to the reference. The problem of polishing a de novo assembly is different, since it involves reads and a reference (i.e. assembly) from the same donor. We expect that the polishing problem comes with its own characteristic bias signals and requires its own methods.

Figure 2: why have authors reported results only about chromosome 20? I agree that it is a comfortable chromosome for development and benchmarking, as it is small enough and an "easy" one, but I would like to see how reference bias affects complex genomic regions as well, like those on chromosome 16, which has one of the highest levels of segmentally duplicated sequence among the human autosomes (https://doi.org/10.1038/nature03187), and/or the short arms of the acrocentric chromosomes, that were resolved for the first time very recently (https://doi.org/10.1126/science.abj6987) and are highlighting interesting heterologous recombination in the human pangenome (https://doi.org/10.1038/s41586-023-05976-y).

As reviewer suggested, we performed the same experiment, with new Q100 project variants, on chromosome 16. We show the results in Supplementary Table S1, and we refer to this result in the main text in the Results subsection titled "Measuring bias across aligners". Remarkably, the results for chromosome 16 (which contains more segmental duplications) are similar to those for chromosome 20.

Typo on Page 6, Line 52: outliers are colored light gray instead of colored light green as reported.

Thanks for pointing it out, we fixed it.

Can I see another Figure that is Figure 2, but stratifying by INDELs length? How does the plot change with INDELs longer than 10/50bps?

Thanks for the suggestion, we added a version of Figure 2 that additionally stratifies by INDEL length. This is the new Supplementary Figure S2. We cite this figure and discuss it in the Results subsection titled "Observations on local bias."

Figures 3 and 4: Why are insertions more affected by reference bias? I see this type of plot all the time, where the insertions have a little more variance with respect to the deletions. Is it because reads carrying insertions are more likely to align incorrectly? Why?

We agree that this needed clearer explanation in the paper text. We have added the explanation of this effect to the result subsection titled "Measuring bias across aligners." Our explanation begins with the sentence “The bias noted for longer insertions seems to be greater than that of longer deletions.”

About the unphased GIAB HG002 VCF, can't authors try to phase those variants and repeat the analysis? There are lots of HiFi reads for HG002, for example, that can be used for the phasing. This would also allow authors to quantify the expected inaccuracy in using the unphased VCF.

We agree that the lack of phasing was a drawback of our previous experiment. In the revised manuscript, we replaced that experiment with a new one that uses a newer, phased VCF generated by the Q100 project. Consistent with that change, we now credit and cite the Q100 project as well as associated projects: the HPRC, T2T and GIAB projects.

Because we were able to switch to this phased HG002 VCF, we deleted the subsection that was titled "VCF inaccuracies and filtering" in the original manuscript. Instead of including that subsection in Results, we added a brief note to the "Context-aware assignment method" subsection within the Methods section stating the potential pitfalls of using an unphased VCF.

In the scan mode evaluation, authors reported that "60% of SNV sites and 67% of gap sites called biased by the NMB-NAB analysis fell inside regions called biased by scan mode." I was hoping for a bit higher percentages. Have authors investigated the cases that do not fall inside bias regions to understand which characteristics those errors have (for example, sequence content, GC%, local sequence entropy, etc…)? Hopefully, authors should just check and incorporate just a few more properties to be able to catch more biased regions without foreknowledge of HET locations.

We appreciate this observation; unfortunately, we have not been able to find any obvious additional features that lead to higher percentages. That said, the percentages in the revised manuscript improved once we switched to using the phased VCF from the Q100 project. Using this more complete VCF file, we get higher ratio of biased sites (75% of the SNVs and 78% of the gaps) falling inside regions called biased by scan mode.

Biastools works on datasets from diploid individuals. What about the plans to extend it in order to work in a haploid setting? And polyploidy? Why did authors restrict to diploidy only?

We agree that more discussion of this point is needed. We added a new paragraph to the Discussion to discuss this point. The paragraph begins with the sentence "Currently, biastools supports only diploid genomes, since most of the work on reference bias avoidance has focused on human and other diploid genomes."

The work shows biastools usage with short reads. Can I already apply biastools on long-read alignments in its current state?

We think the question of how reference bias manifests with long reads is not yet fully answered. Here we have explicitly focused on short reads, as we explain at the beginning of the Results section. ("We focus on bias in the context of diploid individuals (i.e.~human) being sequenced using high-quality short reads, e.g.~from Illumina instruments.")

We do think that reference bias also exists for long reads, but that it manifests in different ways compared to what biastools is trying to measure. An example of another paper that explores this point is: https://doi.org/10.1038/s41592-023-02069-6.

Probably, it is out of the scope, but how much reference bias affects alignment coming from an all-vs-all comparison of reads/contigs?

If we understand the scenario, reads from one individual ("donor") are being aligned to haploid contigs assembled from a second unrelated individual ("reference"). In this case, yes, there is the potential for reference bias since alleles present in the donor may not be present in the reference.

If we understand the comment correctly, then we agree that this is analogous to the scenarios we do assess in the manuscript. Of course, our manuscript concentrates on the most common scenario, which is alignment to an established haploid reference like CHM13 or GRCh38.

Both reference numbers 13 and 29 have issues with the name "Jouni Sirén".

Thanks for pointing this out, we fixed it.

Reviewer #2: The authors present biastools, a tool for analyzing reference bias in genomic sequencing data. It addresses the known problem in genomic data analysis where reads from non-reference alleles might fail to align correctly to their point of origin. This software can be of interest to the bioinformatic community, allowing users and developers of bioinformatics software to tune and optimize alignment algorithms as well as downstream tools.

Major comments.

1. Providing performance metrics of the tool, such as CPU time and memory usage, would be useful for potential users.

Thanks for the suggestion, we made a new result subsection “Computational Performance” to show the running time and memory usage of different biastools mode compared to sequence alignment stage. Since the biastools –simulate mode is mostly based on Mason 2, we didn’t include it in the result.

2. The discussion mentions that "biastools's methods are applicable to long-read alignments as well." It would be nice to add the validation of this statement, especially considering that long reads are actively used for modern whole-genome assemblies. It is in particular challenging with short reads alone to assess highly repetitive regions such as centromeres in T2T-CHM13 (discussed in 2.4). Also worth noting, T2T-CHM13 assembly contains other long complex repeats besides centromeres that might be worth taking into account for the calculations for Table 4.

Our statement regarding long reads is intentionally nuanced. This is because: while it is true that these same methods could be applied to long-read datasets, we believe that reference bias will manifest differently for long reads, and may ultimately necessitate different or additional methods. We think our paper clearly addresses itself to the main problem that the most readers will encounter in practice, i.e. alignment of short reads to a linear or pangenome reference, a paradigm that dominates non-assembly projects.

3. The proposed approach to the quality assessment is interesting, but its validation lacks a bit of clarity. For example, page 14: "scan mode called 1,676 biased regions on chromosome 20, covering 1.8% of its bases". In total, 447 SNVs and 115 gaps fell inside biased regions, but it is unclear how many of the 1,676 biased regions do not contain gaps or SNVs. Would be good to have information where these regions are located and why they were called "biased".

With the new Q100 VCF file, now we have 3,384 biased regions. Out of all the regions, there are only 78 containing no SNPs or gaps. Notice there are also variants that we didn’t analyze such as homozygous ALT or being ambiguous. Out of the 78 biased regions that contain no SNPs or gaps, 39 of them are in the centromere region (chromosome 20 p11.1 to q11.1). Other cases seem to be regions that are gaining reads from other places. In other words, false variants can be seen in the alignment in those regions.

4. The study would benefit from a comparison with other assembly quality evaluation tools, or at least a clear explanation of how biastools differs in scopes and application from some other reference-based tools. While the direct assessment of "reference bias" is quite unique, there are many different tools for assembly quality assessment providing various informative metrics. Such explanation and comparison with known methods could help readers to better understand how they could use biastools in their practice.

We thank the reviewer for this suggestion. We have looked into the literature on assembly quality assessment. We observe that many common assembly measures are quite unrelated to what we measure in biastools, such as those concerning continuity or completeness.

Base-level correctness measures such as the "base-level error rate" do come somewhat close to, for example, the variant density measure used in biastools' "scan" mode. While we did not find any examples of this in the literature, we also speculated that diploid assemblies could be assessed using something like our "non-diploid score" from "scan" mode. In short, while we think there are some connections, we judge that they are not strong enough to warrant discussion in the manuscript.

Minor comments.

1. Authors could consider the possibility to extend the aligners' comparison with minimap2 as one of the most widely used aligners.

We thank the reviewer for this suggestion. We added Minimap 2 in all of our experiments that used read aligners. We found that, overall, Minimap 2 performed similarly to BWA-MEM, but somewhat worse than BWA-MEM -L 30. We added some new phrases describing these results to the subsection titled "Measuring bias across aligners."

2. The paper contains minor typos and syntax errors. For instance, page 19, "the the individual". Page 16, "We collect all the bias regions improved by 25% in LevioSAM 2, can measure how many improved bases are from the regions near centromere, and how many are not." - this sentence seems to be a bit unclear.

Thanks. We fixed it.

2. It would be convenient to provide a small test dataset that can be used by new users to familiarize with the software.

We added a tutorial directory in the github link, which the user can run the example in a short time

**2^nd^ round**

**Reviewer 1**

The authors addressed my concerns, I don't have any other clever comments! In the generated PDF, the citation 14 still has issues with the name of Siren

**Reviewer 2**

Previous concerns have been addressed by authors.
